# Silver ion-doped CdTe quantum dots as fluorescent probe for Hg^2+^ detection

**DOI:** 10.1039/d0ra07140d

**Published:** 2020-10-23

**Authors:** Huazheng Li, Wangwei Lu, Gaoling Zhao, Bin Song, Jing Zhou, Weixia Dong, Gaorong Han

**Affiliations:** State Key Laboratory of Silicon Materials & School of Materials Science and Engineering, Zhejiang University Hangzhou 310027 P. R. China glzhao@zju.edu.cn; State Key Laboratory of Silicon Materials & Department of Physics, Zhejiang University Hangzhou 310027 P. R. China; Department of Traffic Management Engineering, Zhejiang Police College Hangzhou 310053 P. R. China; School of Materials Science and Engineering, Jingdezhen Ceramic Institute Jingdezhen Jiangxi 333403 P. R. China

## Abstract

Mercury(ii), which is a well-known toxic species, exists in the industrial waste water in many cases. In the present work, CdTe quantum dots (QDs) are studied as a fluorescence probe for Hg^2+^ detection. Ag ions are induced to QDs to enlarge their detection concentration range. l-cysteine is employed in the QD-based fluorescence probe to connect QDs with Hg^2+^. X-ray diffraction, transmission electron microscopy and X-ray photoelectron spectroscopy results indicate the formation of zinc blende CdTe QDs with sizes of ∼5 nm and the existence of Ag^+^ in crystalline CdTe. Photoluminescence (PL) spectra and PL decay spectra were acquired to investigate the emission mechanism of Ag-doped CdTe QDs, revealing multi-emission in QD samples with higher concentrations of Ag^+^ doping. The highest PL quantum yield of the QD samples was 59.4%. Furthermore, the relationship between the fluorescence intensity and the concentration of Hg^2+^ has been established. Two linear relationships were obtained for the plot of F/F_0_ against Hg^2+^ concentration, enlarging the detection concentration range of Hg^2+^.

## Abstract 2

Ag-doped CdTe QDs emit multiple-fluorescence peaks, and the relationship between fluorescence intensity and the concentration of Hg^2+^ is established. Two linear relationships are obtained, which is benefit to the extension of detection range.

## Graphical abstract

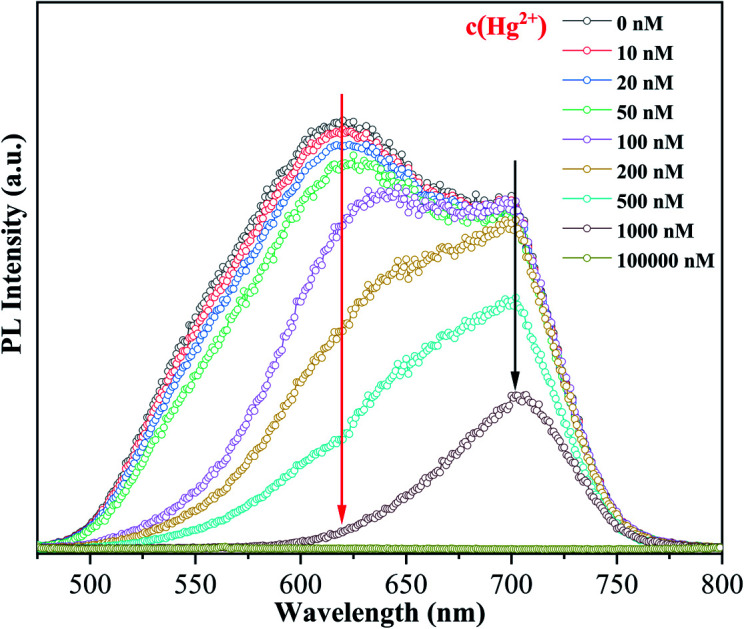

## 1. Introduction

Mercury, which is contained in almost all minerals and plays an important role in many products, is widely distributed in nature. However, mercury(ii) is a toxic heavy metal ion which has a great negative impact on human health and the ecological environment. Therefore, many analytical methods, such as analytical spectroscopic methods^1–3^ and fluorescence methods,^4–6^ have been developed for discriminating and detecting toxic mercury(ii) ions. Analytical spectroscopic methods, including atomic absorption spectrometry,^1^ inductively coupled plasma mass spectrometry,^2^ and inductively coupled plasma optical emission spectrometry,^3^ have already been used for accurate discrimination and detection of heavy metal ions. However, most of these methods are high-cost, are time-consuming, or require complicated sample pretreatment or large sample volumes in the analytical process. Therefore, it is of great importance to develop a rapid and smart method with high precision for Hg^2+^ detection. Fluorescence analytical method^4–6^ as one of the simplest and effective methods has attracted wide attentions, as researchers have found that photoluminescence can be affected by the ionic environment. Fluorescent organic dyes^7,8^ are a group of commonly used fluorescence probes; due to their high selectivity, they have already been applied for Hg^2+^ detection. Other organics, such as G-quadruplex DNAzymes^9^ and naphthalimide derivatives,^10^ have also been developed for Hg^2+^ detection. However, the application of fluorescent dyes in probes is limited in some fields because of their disadvantages, such as low fluorescence intensity and poor light stability.

On the other hand, QDs are promising candidates for ion detection due to their size-dependent fluorescence emissions.^11–14^ The QD size is inversely proportional to the excited state energy; therefore, larger-sized QDs result in lower energy, which consequently changes the emission wavelength. In addition, QDs possess many other unique properties that match well with metal ion detection, such as a high quantum yield and long fluorescence emission lifetime.^15,16^ Hence, QDs have already been widely studied for detection of heavy metal ions. Ions such as cadmium(ii) and zinc(ii) can increase the luminescence of Cd-based QDs, whereas copper(ii) and mercury(ii) ions will quench the emission intensity.^17^ Recently, Wu's group^18^ used a ratiometric fluorescence sensor of metal–organic frameworks (MOFs) and CdTe QDs to detect Hg^2+^ and Cu^2+^ under the UV lamp, Zhang's group^19^ embedded CdTe:Zn QDs into a silica core as energy donors, and a rhodamine B derivative was linked on the particle surface to detect Hg^2+^. Furthermore, the selectivity of the analytical method can be improved through surface modification of QDs. The specific interaction between Hg^2+^ and the N atoms of amino groups as well as carboxyl groups in l-cysteine can selectively capture Hg^2+^ to form amino-Hg^2+^-carboxyl base pairs,^20^ which verifies that high selectivity exists between Hg^2+^ and N atoms and can be used to distinguish Hg^2+^ from other heavy metal ions.

CdTe QDs have good chemical stability, good light stability, adjustable emission wavelengths, and good water solubility, and they have been used for the detection of Hg^2+^;^21,22^ however, their detection range is limited, and their accuracy decreases at high Hg^2+^ concentration. Doping is an effective way to modify the intrinsic properties of corresponding QDs, such as their light capture capability and fluorescence properties.^23–25^ In addition, a long excited state lifetime is a key factor in the design of efficient fluorescence probes; it is conducive to preventing the interference effects of other excitation signals. Silver is beneficial to improvement of the fluorescence emission lifetime^26–28^ and intensity.^29,30^ To date, however, no research has been reported on Ag-doped CdTe QDs fluorescence probes for Hg^2+^ detection.

In order to enlarge the detection range of CdTe QDs-based fluorescent probes for Hg^2+^, in the present work, we prepared Ag-doped CdTe QDs by a low-cost and facile aqueous phase method. The effects of Ag doping on the photoluminescence properties and emission mechanisms were investigated. Multi-emission mechanisms occur for the CdTe QDs with higher concentrations of Ag-doping, which demonstrate higher quantum yields. l-cysteine was chosen as a surface ligand to improve the capture ability of Hg^2+^. The higher quantum yield Ag-doped CdTe QDs were employed to obtain fluorescent probes for Hg^2+^ detection. The relationship between the fluorescence intensity of the probes and the concentration of Hg^2+^ was studied.

## 2. Experimental methods

### 2.1. Synthesis of the pure CdTe QDs and Ag-doped CdTe QDs

Firstly, the tellurium precursor solution was synthesized by dissolving sodium borohydride (10 mmol) and tellurium powder (4 mmol) in 2.5 mL distilled water, followed by a reaction at 30 °C in nitrogen atmosphere for 3 h. The mixture of Cd^2+^-MPA and Ag^+^-MPA was prepared by dissolving Cd(NO_3_)_2_, AgNO_3_ (*n*_Cd_ + *n*_Ag_ = 4 mmol) and mercaptopropionic acid (MPA, 8 mmol) in distilled water. In order to adjust the prepared mixture solution to an appropriate pH value, NaOH solution (1 M) was added dropwise up to pH 6. Then, the mixture was treated in a 250 mL three-neck round-bottom flask at 80 °C under nitrogen atmosphere for half an hour. Finally, all of the as-prepared tellurium precursor solution was rapidly injected into the three-neck round-bottom flask and reacted with the former mixture at 100 °C. The Ag-doped CdTe QDs were obtained by centrifugation. As a comparison, pure CdTe QDs were also prepared using the same process.

### 2.2. Synthesis of the l-cysteine-capped Ag-doped CdTe QDs

The fluorescence probe was obtained by replacing the initial stabilizer (MPA) with l-cysteine. In a typical procedure, l-cysteine (0.2 mmol) was dissolved in 10 mL deionized water. The l-cysteine solution was then added to 0.05 mmol Ag-doped CdTe QDs and stirred for 40 min. The samples were obtained by centrifugation and redissolved in deionized water for further use in the next step.

In order to measure the stability of the Ag-doped CdTe QDs at different pH values, Ag-doped CdTe QDs were redissolved in deionized water with different pH values (adjusted by NaOH and H_2_SO_4_), and their performance was measured after 1 hour of aging.

### 2.3. Characterization

Powder X-ray diffraction (XRD) was used to characterize the phases and crystallinities of the samples. Data were collected on a X'Pert PRO X-ray diffractometer with Cu Kα radiation (*λ* = 1.54178 Å) at a beam current of 40 mA. DI X-ray photoelectron spectroscopy (XPS) was used to analyze the surface composition. Transmission electron microscopy (TEM) carried out on a Tecnai F20 was used to verify the morphologies and microstructures of the samples. Photoluminescence (PL) spectra and photoluminescence decay measurements were achieved by a FLS920 fluorescence emission spectrophotometer under a 405 nm laser.

## 3. Results and discussion

### 3.1. Microstructure of Ag-doped CdTe quantum dots

**Fig. 1:**
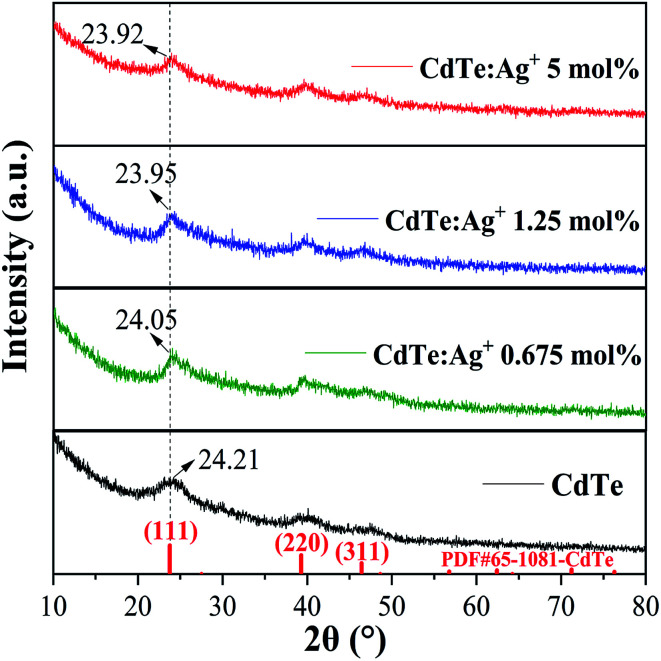
XRD patterns of the pure CdTe QDs and Ag-doped CdTe QDs prepared with 0.675, 1.25 and 5 mol% Ag precursor.

Fig. 1 shows the XRD patterns of the pure CdTe QDs and the Ag-doped CdTe QDs prepared with various concentrations of Ag precursor. All the peaks of the samples correspond to CdTe zinc blende (PDF#65-1081). No peaks related to metallic Ag can be observed for the samples, indicating that Ag does not exist in the form of particles or clusters when the Ag ions are introduced. The diffraction peaks of the samples located at 2*θ* = 24.21°, 39.95° and 46.91° can be assigned to the (111), (220) and (311) facets of CdTe, respectively. As shown in Fig. 1, the peak at 2*θ* = 24.21° shifts to 24.05°, 23.95° and 23.92° with increasing Ag^+^ precursor concentration from 0 to 0.625, 1.25 and 5 mol%, respectively, indicating that the introduction of Ag ions induced a larger lattice constant of the samples. This can be ascribed to the larger radius of Ag^+^ (1.15 Å)^27,31^ than of Cd^2+^ (0.97 Å).^32^

**Fig. 2:**
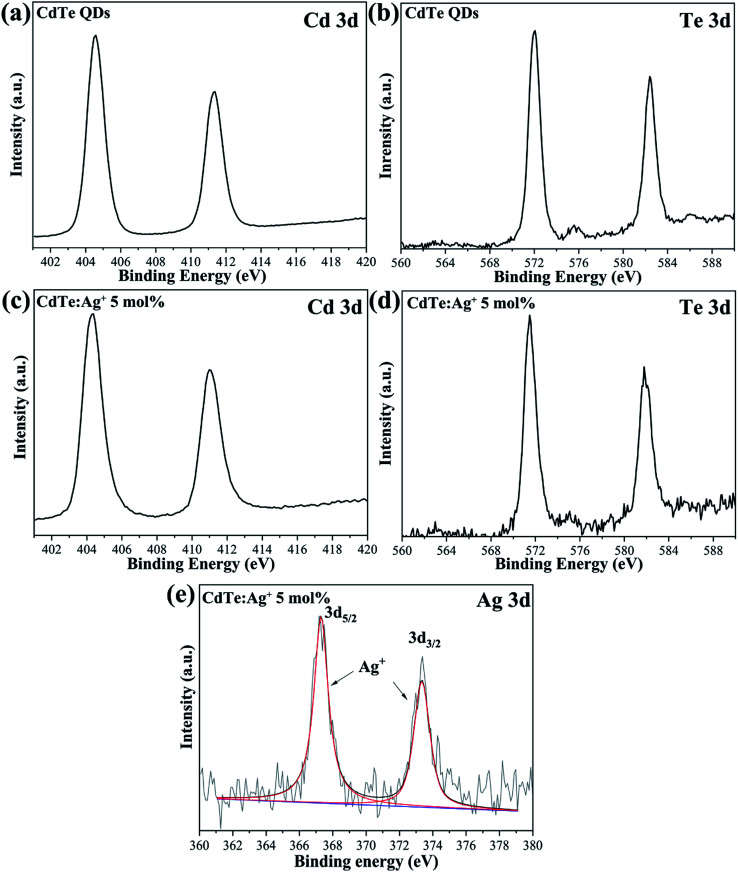
XPS of the pure CdTe QDs (a and b) and the Ag-doped CdTe QDs prepared with 5 mol% Ag precursor (c–e).

Fig. 2 shows the typical XPS spectra of the pure CdTe QDs and the Ag-doped CdTe QDs prepared with 5 mol% Ag precursor. The features at 404.9 and 411.6 eV in Fig. 2a and 572.0 and at 582.4 eV in Fig. 2b for the pure CdTe QDs can be assigned to Cd 3d and Te 3d, respectively.^33,34^ In Fig. 2c and d, the binding energies of the Cd and Te species are 404.5 and 411.2 eV and 571.5 and 582.0 eV, respectively. Fig. 2e shows the high-resolution binding energy spectra of the Ag species; the peaks observed at 367.4 and 373.8 eV can be assigned to Ag 3d,^30^ indicating that Ag is monovalent in the samples. Compared with the pure CdTe QDs, the binding energies of the Cd and Te species for the Ag-doped CdTe QDs shifted to lower positions, indicating that Ag^+^ was successfully doped in the CdTe QDs.

**Fig. 3:**
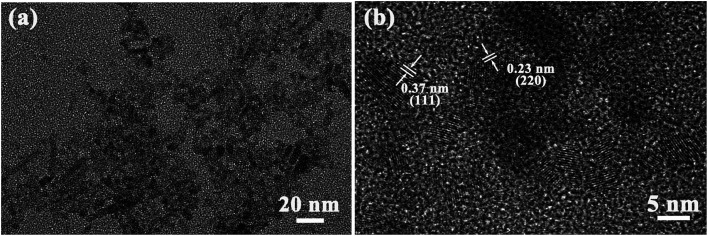
TEM (a) and HRTEM (b) images of the Ag-doped CdTe QDs prepared with 5 mol% Ag precursor.

Fig. 3 shows TEM and HRTEM images of Ag-doped CdTe QDs prepared with 5 mol% Ag precursor. As shown in Fig. 3a, the QDs present ellipsoidal shapes and the size of the QDs is about 5 nm, which is smaller than the Bohr exciton radius (∼7 nm (ref. 35)). The corresponding HRTEM images (Fig. 3b) exhibit the (111) and (220) facets of CdTe zinc blende, which is consistent with the XRD pattern (see Fig. 1). The introduction of Ag^+^ in the CdTe QDs does not change the structure or morphology of the CdTe QDs.

### 3.2. Photoluminescence properties and mechanism of Ag-doped CdTe quantum dots

**Fig. 4:**
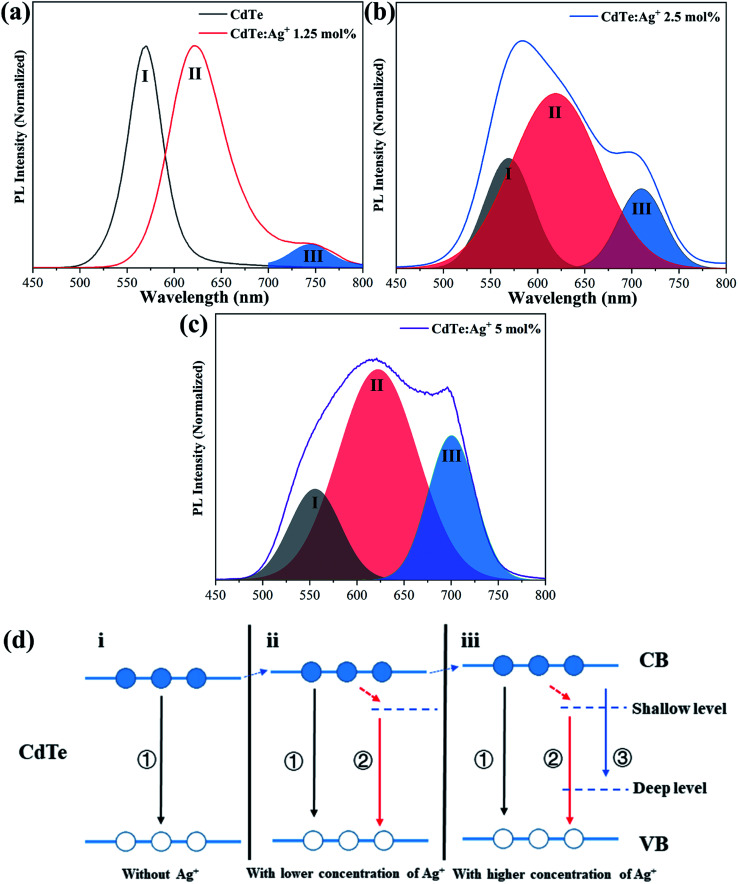
Photoluminescence spectra, de-convolutions and Gauss fits of the pure CdTe QDs and the Ag-doped CdTe QDs with various concentrations of Ag precursor (a–c); schematic of the band structure of the CdTe QDs and the emission process (d).

Fig. 4a–c show the PL spectra and the de-convolutions and Gauss fits of the pure CdTe QDs and the Ag-doped CdTe QDs with various concentrations of Ag precursor. The emission peak of the pure CdTe QDs is at 570 nm. The emission peak shifts to 620 nm and a shoulder appears at 744 nm when 1.25 mol% Ag is added to the precursor (Fig. 4a). Further increase of the concentration of Ag precursor results in a broad emission band with a shoulder around 700 nm and a peak at a shorter wavelength (see Fig. 4b and c), indicating that multi-emission occurs in these samples. De-convolutions and Gauss fitting for the emission bands in Fig. 4b and c revealed three characteristic peaks, which are labeled as I, II, III, respectively (in Fig. 4a–c). Peak I shifts from 570 nm to 565 nm and 555 nm when the Ag content in the precursor increases from 0 mol% to 2.5 mol% and 5 mol%, respectively. Peak II located at 620 nm does not change with increasing Ag amount in the precursor. Peak III shifts from 744 nm to 711 nm and 702 nm with increasing Ag content in the precursor from 1.25 mol% to 2.5 mol% and 5 mol%, respectively.

The evolution of the photoluminescence spectra with Ag concentration in the precursor can be explained based on the different photoluminescence mechanisms for various Ag concentrations in the precursor. Combined with the present XRD results (see Fig. 1) and previous work,^36^ Ag enters the CdTe crystal by replacing Cd^2+^ and/or as interstitial ions. Accordingly, the band structures and photoluminescence mechanisms of the Ag-doped CdTe QDs are suggested in Fig. 4d. When a few Ag^+^ ions are doped in the CdTe QDs, Ag^+^ may replace Cd^2+^ and result in (1) enlarging the lattice constant, thus raising the lowest energy level of the conduction band,^30,36,37^ and (2) introducing anion vacancies, thus forming a shallow level under the conduction band of CdTe^38,39^ (see Fig. 4d-ii). When the number of Ag^+^ ions increases, some Ag^+^ may replace Cd^2+^ and other Ag^+^ ions may occupy interstitial positions in the crystal. Therefore, introduction of Ag^+^ in this case not only raises the conduction band and forms a shallow level but also produces a deep level in the bandgap^36,38^ (see Fig. 4d-iii). Based on the band structure in Fig. 4d, the possible photoluminescence mechanisms can be listed as follows. (I) The excited electrons in the conduction band transfer to the valence band of CdTe and emit photons; (II) the excited electrons in the conduction band relax to the shallow level and then transfer to the valence band and emit photons; (III) the excited electrons transfer from the conduction band to the deep level. The pure CdTe QDs show an emission peak at 570 nm (Peak I) caused by the recombination between electrons in the conduction band and holes in the valence band. When 1.25 mol% Ag^+^ is added to the precursor, the sample shows two emission peaks at 620 nm (Peak II) and 744 nm (Peak III), resulting from the electron transfer from the shallow level to the valence band and the electron transfer from the conduction band to the deep level, respectively. The samples with 2.5 mol% and 5 mol% Ag^+^ in the precursor undergo tri-recombination mechanisms, as illustrated in Fig. 4d; thus, both samples show three photoluminescence peaks. Among these peaks, Peak I and Peak III show blue shifts with increasing Ag^+^ amount in the precursor, which can be ascribed to the increasing conduction band with increasing Ag^+^ amount in the precursor.

**Fig. 5:**
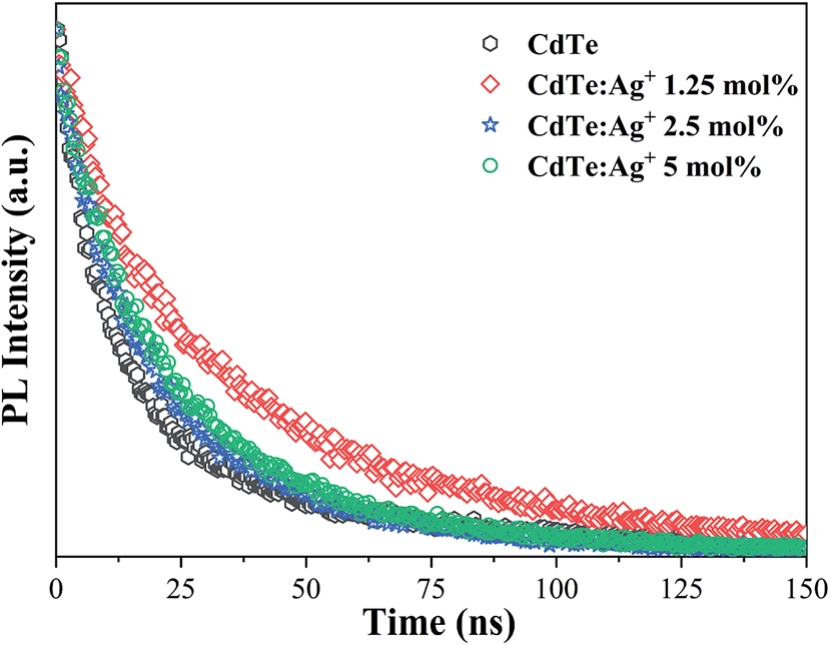
PL decay spectra of the pure CdTe QDs and the Ag-doped CdTe QDs with various concentrations of Ag precursor: 1.25 mol%, 2.5 mol% and 5 mol%.

Fig. 5 shows the PL decay spectra of the pure CdTe QDs and Ag-doped CdTe QDs prepared with various amounts of Ag precursor. The PL intensity data in Fig. 5 were measured at 570 nm for the sample with pure CdTe QDs and at 620 nm for the Ag-doped samples (1.25 mol%, 2.5 mol% and 5 mol% Ag^+^ in the precursor). Compared with the pure CdTe QDs, all the Ag-doped CdTe QDs need more time to decay to the same intensity, indicating that Ag-doping improves the fluorescence lifetime. The fluorescence lifetime of the sample reaches an optimum value when 1.25 mol% Ag is added to the precursor, indicating that the electrons at the shallow level have relatively long lifetimes. This evolution of the fluorescence lifetime with Ag precursor concentration also confirms the multi-emission mechanism. When the Ag precursor concentration reaches 2.5 mol%, the conduction band causes more emission to transform to the deep level; therefore, the fluorescence lifetime becomes shorter than that of the sample with 1.25 mol% Ag added to the precursor.

The PL quantum yields of the samples were calculated based on the PL data and optical absorbance data using the internal standard method and Rhodamine B as a standard reference. The results showed that the Ag-doped CdTe QDs with 5 mol% Ag precursor possessed a quantum yield of 59.4%, which is much higher than that of the pure CdTe QDs, *i.e.* 20.3%. The enhancement of the quantum yield can be ascribed to the shallow level as well as the deep level caused by Ag doping, which extend the PL lifetime and increase the chance of emission.

### 3.3. Ag-doped CdTe QDs as a fluorescence probe for Hg^2+^ detection

**Fig. 6:**
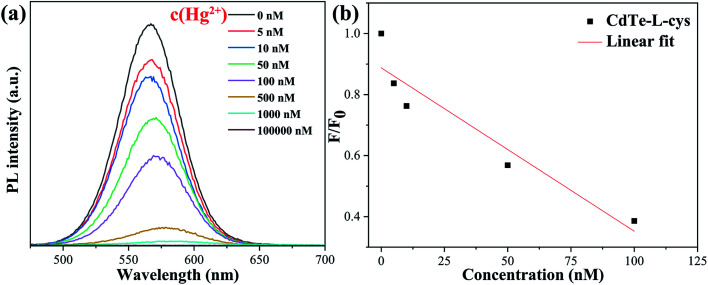
Fluorescence emission spectra of the pure CdTe QDs in the presence of Hg^2+^ at different concentrations (a); relationship between the PL intensity at 570 nm and the concentration of Hg^2+^ (b).

Fig. 6a shows the fluorescence emission spectra of the pure CdTe QDs in the presence of Hg^2+^ at various concentrations. The PL intensity decreases with increasing Hg^2+^ concentration. The PL intensity is absolutely quenched as the Hg^2+^ concentration increases to 1000 nM, indicating that the pure CdTe QDs probe is not satisfactory for detection of mercury ions with high concentration. For a more intuitive display, Fig. 6b shows the relationship between the PL intensity and Hg^2+^ concentration. F refers to the PL peak intensity of the QD sample in Hg^2+^ solution and F_0_ refers to the PL peak intensity of the QD sample in solution without Hg^2+^ (blank line in Fig. 6a).

**Fig. 7:**
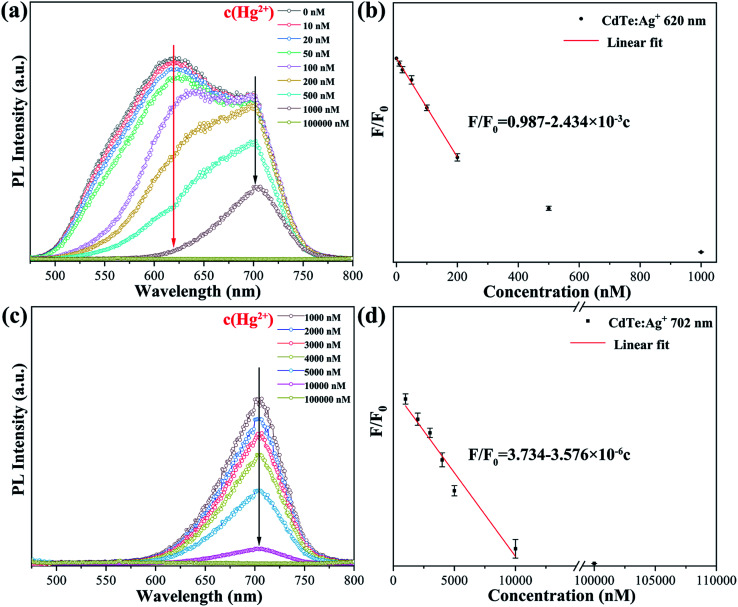
Fluorescence emission spectra of the Ag-doped CdTe QDs prepared with 5 mol% Ag precursor in the presence of Hg^2+^ with various concentrations (a and c); relationship between *F*/*F*_0_ at 620 nm and 702 nm with the concentration of Hg^2+^ (b: 620 nm, d: 702 nm).

As revealed by the photoluminescence study of the Ag-doped CdTe QDs, the sample with 5 mol% Ag precursor demonstrates a high PL quantum yield of 59.4% and broad emission bands with strong intensity at 620 nm (peak II) and 702 nm (peak III) (see Fig. 4c). The high PL quantum yield enables a small amount of the sample to emit strong PL intensity; thus, the fluorescence change is more obvious. The broad emission band is expected to extend the detection concentration range. Therefore, the QD sample with 5 mol% Ag precursor should be a promising candidate for a fluorescence probe with a larger detecting range. Fig. 7 shows the fluorescence emission spectra of the Ag-doped CdTe QDs prepared with 5 mol% Ag precursor in the presence of Hg^2+^ at various concentrations. The PL intensity of the peak at 620 nm decreases continuously with increasing Hg^2+^ concentration [10–200 nM] (the red arrow in Fig. 7a). Meanwhile, the PL intensity of the peak at 702 nm remains almost unchanged at low Hg^2+^ concentration. Moreover, the fluorescence of the peak at 620 nm is almost quenched when the concentration of Hg^2+^ reaches 1000 nM. On the other hand, the PL intensity of the peak at 702 nm decreases continuously with further increase of the Hg^2+^ concentration [1000–100 000 nM] (Fig. 7c).

Fig. 7b and d show the plots of *F*/*F*_0_ against Hg^2+^ concentration, and the fitted linear data can be expressed as eqn (1) and (2) at 620 nm and 702 nm, respectively.

1 *F*/*F*_0_ = 0.987 − 2.434 × 10^−3^*c*

2 *F*/*F*_0_ = 3.734 − 3.576 × 10^−6^*c*

where *c* represents the concentration of Hg^2+^. Therefore, the detection range has been extended to two areas, not only a low concentration range [10–200 nM] but also a high concentration range of Hg^2+^ [1000–100 000 nM], and both have high accuracy.

**Fig. 8:**
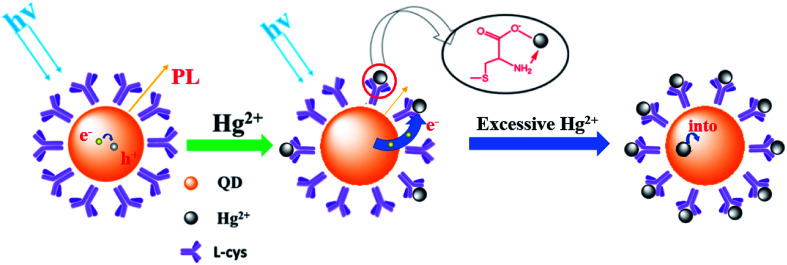
Schematic of the process of the Ag-doped CdTe QDs fluorescence probe for Hg^2+^ detection.

Fig. 8 shows a schematic of the mechanism of the Ag-doped CdTe QDs fluorescence probe for Hg^2+^ detection. l-cysteine contains both amino and carboxyl groups; thus, it forms a strong bond with Hg^2+^. Therefore, the quenching of the fluorescence of the l-cysteine-capped QDs by Hg^2+^ can be attributed to electron transfer from the QDs to Hg^2+^. The introduction of Ag^+^ induces multi-emission mechanisms and results in multiple fluorescence peaks. At low concentrations of Hg^2+^, Hg^2+^ is connected to the surface ligands of the CdTe QDs and electrons from the shallow energy level more easily transfer to the Hg^2+^, resulting in a decrease in the fluorescence at 620 nm. At high concentrations of Hg^2+^, the Hg^2+^ will enter the CdTe QD crystals, destroying the energy level structure of the Ag-doped CdTe QDs and causing the fluorescence at 702 nm to continue to decline (see Fig. 7). Thus, the relationship between the fluorescence intensity and the concentration of Hg^2+^ is established: *F*/*F*_0_ = 0.987 − 2.434 × 10^−3^*c* [0–200 nM] and *F*/*F*_0_ = 3.734 − 3.576 × 10^−6^*c* [1000–100 000 nM]. Mercury ions can be detected accurately in these two concentration ranges.

**Fig. 9:**
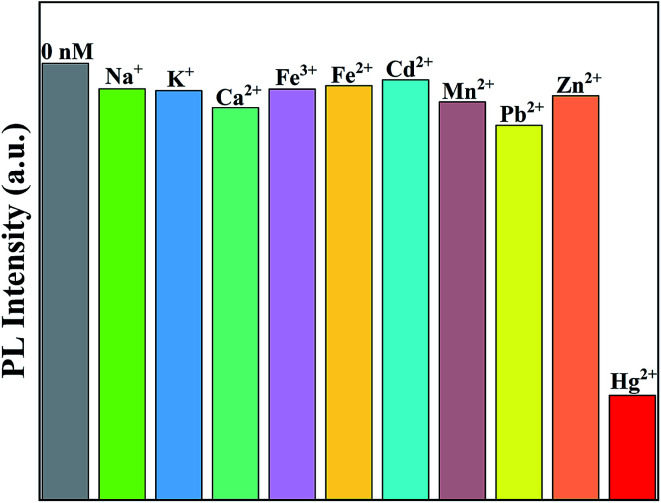
The strongest fluorescence intensities of the Ag-doped CdTe QDs prepared with 5 mol% Ag precursor after detecting various metal ions (1000 nM) as a fluorescent probe.

In order to eliminate the interference effects of common ions such as Na^+^, K^+^, Ca^2+^, Fe^3+^, Cd^2+^, Mn^2+^, Pb^2+^ and Zn^2+^ in domestic water systems, the effects of metal ions other than Hg^2+^ on the fluorescence intensity of the Ag-doped CdTe QDs prepared with 5 mol% Ag precursor were investigated. Fig. 9 shows the strongest fluorescence intensity of the Ag-doped CdTe QDs prepared with 5 mol% Ag precursor after detecting various metal ions as a fluorescence probe. The fluorescence intensity decreases slightly in the presence of Na^+^, K^+^, Ca^2+^, Fe^3+^, Cd^2+^, Mn^2+^, Pb^2+^ and Zn^2+^ (1000 nM), indicating weak effects of these ions on the fluorescence emission process. Therefore, the Ag-doped CdTe QDs fluorescence probe has high sensitivity to Hg^2+^.

**Fig. 10:**
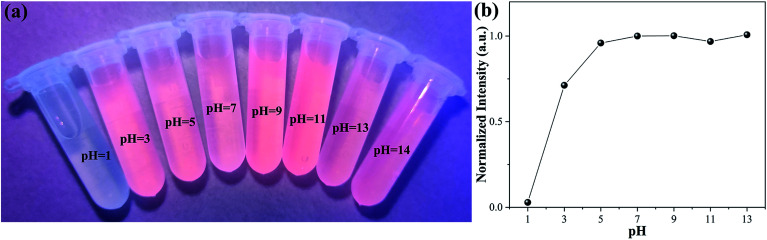
Photograph of Ag-doped CdTe QDs prepared with 5 mol% Ag precursor dispersed in various pH solutions under UV light irradiation (a). The normalized PL intensities of the Ag-doped CdTe QDs prepared with 5 mol% Ag precursor for detecting Hg^2+^ (1000 nM) at various pH values (b).

Fig. 10a shows a photograph of the Ag-doped CdTe QDs prepared with 5 mol% Ag precursor dispersed in various pH solutions under UV light irradiation. When the pH ≥ 3, the Ag-doped CdTe QDs dispersed in solution all emit fluorescence, and the intensity is almost the same at various pH values; this indicates that the Ag-doped CdTe QDs have good pH stability when the pH ≥ 3. Fig. 10b shows the normalized PL intensity of Ag-doped CdTe QDs prepared with 5 mol% Ag precursor for detecting Hg^2+^ (1000 nM) at various pH values. The intensity is basically maintained at the same value; however, when the pH is 3, the intensity drops significantly, indicating that the Ag-doped CdTe QDs are unstable at low pH values. However, as a fluorescent probe, the Ag-doped CdTe QDs still have high stability and accuracy at pH ≥ 5. According to the studies of the effects of pH value and ion interference, the Ag-doped CdTe QDs can be applied in a variety of environments.

## 4. Conclusion

Ag-doped CdTe QDs were prepared by an aqueous phase method. With increasing Ag doping concentration, shallow and deep energy levels were introduced in the energy level structure of CdTe, resulting in multiple fluorescence peaks. Capped by l-cysteine, the sample with 5 mol% Ag precursor was used as a fluorescent probe to detect Hg^2+^; Hg^2+^ has different quenching mechanisms for different fluorescence emission peaks, and the detection linear ranges were 10 nM to 200 nM and 1000 nM to 100 000 nM. Two linear relations, caused by declining of the fluorescence emission peak intensity, extended the detection range. The results showed that the l-cysteine capped Ag-doped CdTe QDs have potential applications in the field of Hg^2+^ detection.

## Conflicts of interest

The authors declare no conflicts of interest in relation to this paper.

## Supplementary Material
